# Supplementary material for: Light controlled 3D micromotors powered by bacteria
Source: Nat Commun. 2017 Jun 28;8:15974. doi: 10.1038/ncomms15974 (PMC5493761; doi:10.1038/ncomms15974)
Supplement: Supplementary Information [file ncomms15974-s1.pdf]

File name: Supplementary Information
Description: Supplementary Figure

File name: Peer Review File
Description:

File name: Supplementary Movie 1
Description: Array of 16 microrotors actuated by smooth swimming bacteria. The movie switches from bright field mode to epifluorescence revealing the high occupancy number of microchambers.

File name: Supplementary Movie 2
Description: Zoomed view of a fully loaded micromotor pushed by 15 bacteria as revealed in the second half of the video where we switch to epifluorescence.

File name: Supplementary Movie 3
Description: A dense array of 36 microrotors all spinning with a smooth and uniform speed.

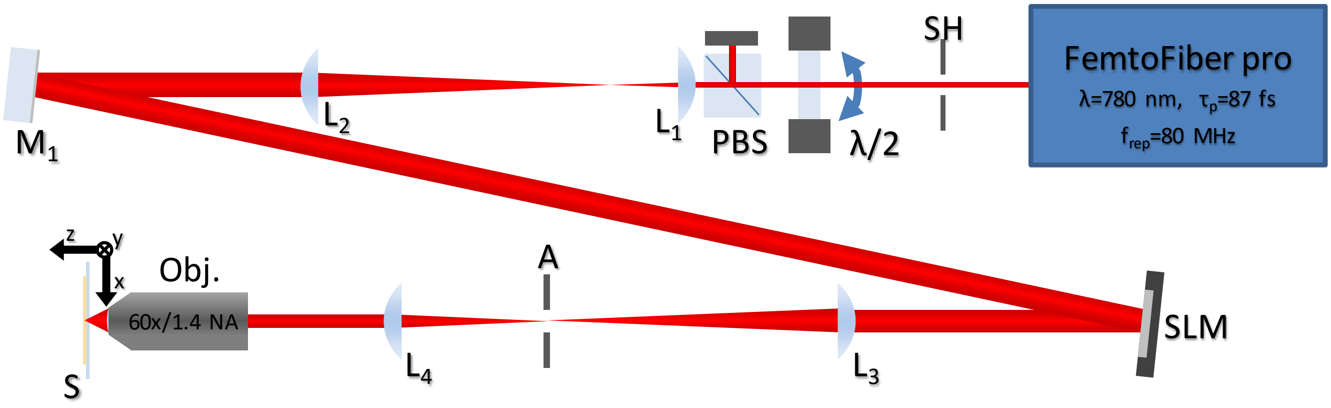

Supplementary Figure 1: Schematic layout of the custom built two-photon polymerization setup. Exposure during fabrication is toggled by an optical shutter (SH). The fabrication laser power is set by a rotatable half-wave plate followed by a polarizing beam splitter cube (PBS). After expansion by lenses L1 and L2 the laser beam is reflected onto a holographic spatial light modulator (SLM), which is in 4f conjugation to the back focal plane of a high numerical aperture oil immersion objective by lenses L3 and L4. The SLM is used to generate multiple fabrication foci and to impose wavefront correction on the fabrication beam. The zero and the high diffraction orders can be blocked in the focal plane of L3 by a thin wire and by an adjustable rectangular aperture (A). During fabrication the high NA focus of the laser is scanned inside a photoresist layer (S) carried on a microscope coverglass. Scanning is done by a 3-axis piezo translation stage (P563.3CD, Physik Instrumente (PI) GmbH & Co. KG) controlled through a NI-DAQ DA card.
